# Transient Left Bundle Branch Block in the Setting of Hyperkalemia

**DOI:** 10.7759/cureus.16602

**Published:** 2021-07-24

**Authors:** Abida Naz, Tikal Kansara, Marco Ruiz Santillan, Mohammad Saeed

**Affiliations:** 1 Internal Medicine, New York Medical College, NYC Health + Hospitals/Metropolitan, New York, USA; 2 Internal Medicine, New York Medical College, Metropolitan Hospital Center, New York, USA

**Keywords:** left bundle branch block, severe hyperkalemia, myocardial infarction, hemodialysis, hyperkalemia-induced ekg changes

## Abstract

Hyperkalemia is a potentially life-threatening condition that can lead to sudden cardiac death. We report a case of transient left bundle branch block (LBBB) pattern on an electrocardiogram (EKG) secondary to hyperkalemia in a patient with a history of end-stage renal disease. A 48-year-old female presented to the emergency department (ED) with chief complaints of weakness and shortness of breath after a missed hemodialysis session. A 12-lead EKG in the ED showed the LBBB pattern with left axis deviation, prolonged PR interval, and peaked T-waves in the precordial leads. The initial serum potassium level was 8.5 mEq/L. EKG changes resolved after correcting the serum potassium level.

## Introduction

Hyperkalemia is a frequent clinical problem most often caused by impaired urinary potassium excretion secondary to acute kidney injury or chronic kidney disease. Severe hyperkalemia is a medical emergency requiring urgent interventions and can lead to life-threatening cardiac arrhythmias if left untreated. Although serum potassium concentration is the most reliable method for determining the severity of hyperkalemia, electrocardiogram (EKG) changes associated with hyperkalemia may suggest the diagnosis before conducting a blood test. Although various EKG changes may be associated with hyperkalemia, peaked T-waves in the precordial leads are usually the first and most commonly recognized findings. Cardiac conduction abnormalities have also been reported with hyperkalemia at severely elevated levels [1,2]. Here, we report a case of transient left bundle branch block (LBBB) secondary to hyperkalemia in a patient with a history of end-stage renal disease (ESRD).

## Case presentation

**Figure 1:**
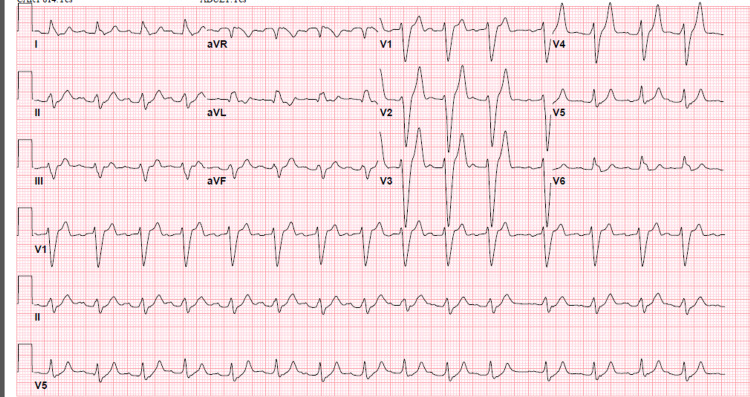
Initial EKG. First-degree atrioventricular block (PR: 222 ms), LBBB (QRS: 174 ms), and tall tented T-waves. EKG: electrocardiogram; LBBB: left bundle branch block

A 48-year-old female with ESRD on hemodialysis presented to the emergency department (ED) with complaints of generalized weakness and shortness of breath after she missed one hemodialysis session. On arrival to the ED, her heart rate was 110 beats per minute, blood pressure was 210/90 mmHg, respiratory rate was 22 breaths per minute, and oxygen saturation was 90% on 2 L of oxygen. General and systemic examinations were suggestive of volume overload. The laboratory analysis showed severe hyperkalemia with a serum potassium level of 8.5 mEq/L, blood urea nitrogen of 118 mg/dL, creatinine of 13 mg/dL, sodium of 134 mEq/L, chloride of 99 mEq/L, bicarbonate of 22 mmol/L, and calcium of 9.4 mg/dL. Urine toxicology was negative. Her initial EKG showed a heart rate of 92 beats per minute, PR interval of 222 ms, QRS duration of 174 ms, dominant S-wave in V1, broad monophasic R-wave in lateral leads, absence of Q-wave in lateral leads (I, aVL, V5-V6), and left axis deviation, suggestive of LBBB (Figure 1). On review of records, a normal baseline EKG with no structural heart disease was noted. Although new-onset LBBB could have been due to myocardial infarction/ischemia or hyperkalemia, due to a history of ESRD with missed hemodialysis, we treated this as a case of severe hyperkalemia which was confirmed with serum potassium level.

**Figure 2:**
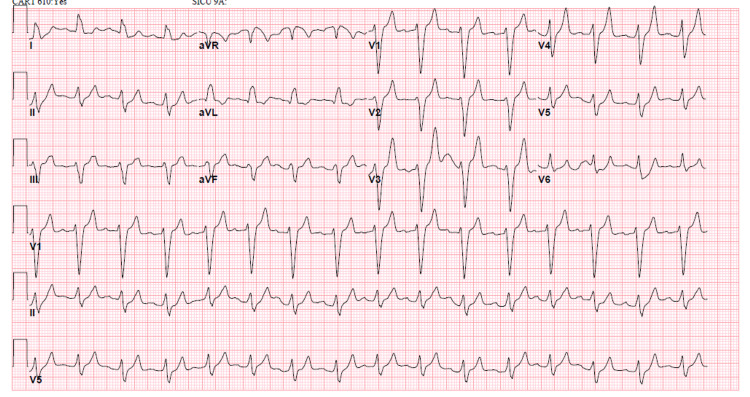
EKG after intravenous calcium gluconate. EKG after calcium gluconate showed shortening of the PR interval (202 ms from 222 ms) and mild narrowing of the QRS complex (158 ms from 174 ms). EKG: electrocardiogram

**Figure 3:**
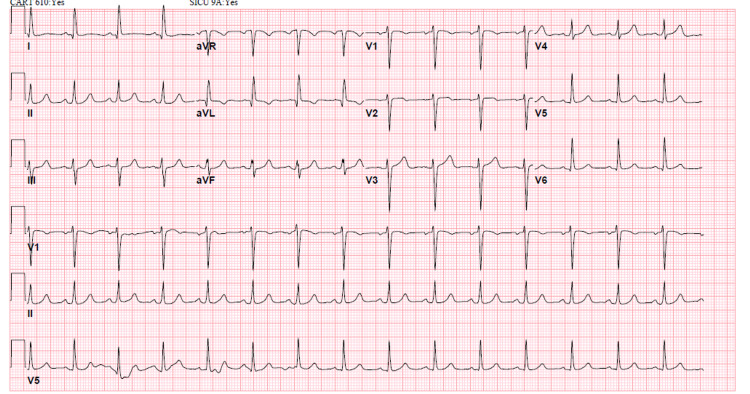
EKG after hemodialysis showing normal sinus rhythm. EKG: electrocardiogram

She was initially treated with intravenous calcium gluconate, insulin with glucose, albuterol nebulization, and intravenous furosemide (Figure 2). Hemodialysis was done on an urgent basis after which potassium level improved to 5.5 mEq/L and her shortness of breath resolved. A repeat EKG showed normal sinus rhythm (Figure 3).

In the setting of ESRD and absence of known precipitating factors for increased serum potassium level, her hyperkalemia was attributed to ESRD. On normal cardiac evaluation including an exercise stress test, her EKG changes were assumed to be related to hyperkalemia which resolved after hemodialysis.

## Discussion

Hyperkalemia may be associated with EKG changes. However, EKG findings in hyperkalemia are seen in only 50-64% of patients with potassium levels of more than 6.5 mEq/L [3]. Sequential EKG changes noted with rising potassium levels include peaked T-waves, prolonged PR interval, prolonged QRS, loss of P-wave, escape rhythms, sine wave configuration, ventricular fibrillation, and pulseless activity or asystole. These EKG changes can be explained by the physiological effect of potassium on myocardial cells. Hyperkalemia decreases the resting membrane potential (RMP), the magnitude of the action potential, and the maximum rate of increase of phase 0 in the cardiac muscle. A high concentration of extracellular potassium slows impulse conduction through all cardiac tissue, accounting for numerous EKG findings. The effect of hyperkalemia depends on the tissue involved, with the atrial myocardium being the most sensitive, the ventricular myocardium less sensitive, and the specialized tissue (sinoatrial node and His bundle) the least sensitive.

Mild-to-moderate hyperkalemia causes depression of conduction between adjacent cardiac myocytes, resulting in the prolongation of the PR and QRS intervals as potassium levels increase. P-wave amplitude disappears early because of the sensitivity of atrial myocytes to hyperkalemia. As the severity of hyperkalemia increases, the sinoatrial and atrioventricular conduction is further suppressed, resulting in the appearance of escape beats and escape rhythms. The QRS complex continues to widen and may blend with the T-wave, creating a sine wave appearance in the EKG. If the potassium level is allowed to rise without treatment, it can cause ventricular fibrillation.

The presence of these EKG findings often guides on how aggressively to treat hyperkalemia [4]. However, there have been reports of normal EKG even with serum potassium levels greater than 10 mEq/L [5]. Adverse outcomes of hyperkalemia include symptomatic bradycardia, ventricular tachycardia, ventricular fibrillation, cardiac arrest, and death. According to one study, the median time from EKG to an adverse event was 47 minutes [4]. Hence, it becomes necessary to treat the patient in the ED as soon as EKG changes are suspicious of hyperkalemia. Previous studies have reported rate-dependent LBBB, wherein the EKG did not show any changes at a lower rate; however, as the heart rate increased, atrial fibrillation with LBBB was seen in a patient with hyperkalemia [6]. As LBBB resolved in our patient after hemodialysis, and cardiac evaluation including exercise stress test was normal, LBBB can be attributed to hyperkalemia.

Severe hyperkalemia with a potassium level of >6.5 mEq/L is regarded as a medical emergency and the patient should receive cardiac monitoring in a controlled setting with immediate medical interventions. If any EKG changes are noted, the patient should be promptly administered membrane stabilizers such as intravenous calcium and cellular potassium uptake agents. This should be followed as soon as possible by therapies to remove potassium from the body, including hemodialysis, diuretics, and gastrointestinal cation exchange resins [7].

## Conclusions

Our case illustrates the effect of serum potassium on EKG which an alert physician can recognize to provide lifesaving treatment even before receiving biochemical confirmation.
